# Urinary and Plasma Levels of Vasohibin-1 Can Predict Renal Functional Deterioration in Patients with Renal Disorders

**DOI:** 10.1371/journal.pone.0096932

**Published:** 2014-06-10

**Authors:** Norikazu Hinamoto, Yohei Maeshima, Daisuke Saito, Hiroko Yamasaki, Katsuyuki Tanabe, Tatsuyo Nasu, Hiroyuki Watatani, Haruyo Ujike, Masaru Kinomura, Hitoshi Sugiyama, Hikaru Sonoda, Yasufumi Sato, Hirofumi Makino

**Affiliations:** 1 Department of Medicine and Clinical Science, Okayama University Graduate School of Medicine, Dentistry and Pharmaceutical Sciences, Okayama, Japan; 2 Department of Chronic Kidney Disease and cardiovascular disease, Okayama University Graduate School of Medicine, Dentistry and Pharmaceutical Sciences, Okayama, Japan; 3 Center for Chronic Kidney Disease and Peritoneal Dialysis, Okayama University Graduate School of Medicine, Dentistry and Pharmaceutical Sciences, Okayama, Japan; 4 Discovery Research Laboratories, Shionogi, Osaka, Japan; 5 Department of Vascular Biology, Institute of Development, Aging, and Cancer, Tohoku University, Sendai, Japan; UNIFESP Federal University of São Paulo, Brazil

## Abstract

Vasohibin-1 (VASH-1) is a negative feedback regulator of angiogenesis, and a small vasohibin-binding protein (SVBP) serves as its secretory chaperone and contributes to its antiangiogenic effects. In the present study, we aimed to define the clinical significance of VASH-1 and SVBP in patients with chronic kidney disease (CKD). We recruited 67 Japanese hospitalized patients with renal disorders with (n = 45) or without (n = 22) renal biopsy samples and 10 Japanese healthy controls. We evaluated the correlations between the plasma and urinary levels of VASH-1/VASH-1-SVBP complex/SVBP and the clinicopathological parameters. The plasma levels of VASH-1 were inversely correlated with age and systolic and diastolic blood pressure and positively correlated with crescent formation. Increased plasma and urinary levels of VASH-1 and VASH-1-SVBP complex were significantly correlated with worse renal outcomes. These results demonstrate an association between elevated urinary and plasma levels of VASH-1 and progressive decline of the renal function, thus suggesting a potential role for VASH-1 in predicting a worse renal prognosis in patients with renal disease, including CKD.

## Introduction

Chronic kidney disease (CKD) is associated with an increased risk of end-stage renal disease (ESRD) and cardiovascular morbidity and mortality. Since the number of patients with ESRD is increasing worldwide, establishing biomarkers to predict renal functional deterioration and prevent the progression of CKD by initiating early medical intervention is required.

Glomerulosclerosis and tubulointerstitial alterations are the most common characteristic histological alterations observed in patients with renal disorders. In general, deterioration of the renal function correlates better with the degree of tubulointerstitial injury than do glomerular alterations [1]. Experimental and biopsy studies have shown that the loss of podocytes [2], [3], [4], [5] and renal capillaries [6] is closely linked with the progression of CKD and renal scarring.

Vasohibin-1 (VASH-1), an endogenous angiogenesis inhibitor, was originally identified in a microarray analysis assessing genes up-regulated by vascular endothelial growth factor (VEGF)-A, a major pro-angiogenic factor [7], in endothelial cells [8]. The human VASH-1 protein is composed of 365 amino acid residues [8] and regulates the proliferation and migration of endothelial cells in an autocrine manner and thus is considered to be a negative feedback regulator of angiogenesis [8]. More recently, the critical role of VASH-1 in the maintenance of endothelial cells against cellular stressors and the regulatory mechanisms of its synthesis via posttranscriptional regulation mediated by the binding of HuR proteins to AU-rich elements in the 3′ untranslated region of VASH-1 mRNAs have been reported [9]. VASH-1 does not contain a classic signal sequence; rather, a small vasohibin-binding protein (SVBP) serves as its secretory chaperon and contributes to its antiangiogenic effects [10]. To date, no cell surface receptors for VASH-1 have been identified. The therapeutic efficacy of VASH-1 in experimental models of tumors, atherosclerosis, proliferative retinopathy [8], [11], [12] and diabetic nephropathy [13], [14] has been reported. However, the circulating and urinary levels of VASH-1 in patients with renal disorders have not been examined to date.

In the present study, we aimed to elucidate the clinical significance of VASH-1 in patients with renal disorders. We determined the plasma, urinary levels of VASH-1 and evaluated the correlations between these parameters and clinical as well as histological variables. This is the first study to examine the clinical significance of VASH-1 in patients with renal disorders, indicating the potential of VASH-1 as a predictive biomarker for progressive renal functional deterioration.

## Results

### Characteristics of the study groups

The mean age of the patients was 50±18 years, the mean estimated glomerular filtration rate (eGFR) was 60.3±34.7 mL/min/1.73 m^2^ and the mean daily level of proteinuria was 2.1±2.9 g/day. The prevalence of hypertension, diabetes mellitus and dyslipidemia was 44%, 22% and 57%, respectively (Table 1). Only the age (51±5 years, 1 unknown), gender (Male 5/Female 4, 1 unknown), and VASH-1-related data were available for the healthy control subjects. The baseline clinical data of healthy controls and classified according to the renal disorder diagnosed by renal biopsies are shown in Table 2. No significant differences were observed among any of the groups. Due to the small number of patients in membranous nephropathy, minimal change, crescentic glomerulonephritis, lupus nephritis, diabetic nephropathy and nephrosclerosis, it was difficult to show the statistical significances among those disorders.

**Table 1:** Clinical parameters of the patients with renal disorders (*n* = 67).

Age	50±18	(17–82)
Gender (male/female)	28/39	
BMI (kg/m^2^)	23.6±3.7	(16.6–33.8)
SBP (mmHg)	130±20	(82–168)
DBP (mmHg)	77±13	(48–107)
Hemoglobin (g/dL)	12.5±2.1	(8.3–16.6)
HbA1c (NGSP) (%)	6.0±1.0	(4.8–9.8)
FPG (mg/dL)	111.8±39.0	(74–331)
T-cho (mg/dL)	227.8±80.7	(106–590)
LDL-C (mg/dL)	132.2±59.1	(29–417)
HDL-C (mg/dL)	60.1±24.5	(27–170)
TG (mg/dL)	149.4±87.5	(42–476)
eGFR (mL/min/1.73 m^2^)	60.3±34.7	(6.1–132.4)
Daily proteinuria (g/day)	2.1±2.9	(0.0–13.1)
Hypertension (%)	43.9	
Diabetes mellitus (%)	22.2	
Dyslipidemia (%)	56.5	

Abbreviations: BMI, body mass index; DBP, diastolic blood pressure; eGFR, estimated glomerular filtration rate; FPG, fasting plasma glucose; HDL-C, high-density lipoprotein cholesterol; LDL-C, low-density lipoprotein cholesterol; NGSP, national glycohemoglobin standardization program; SBP, systolic blood pressure; T-cho, total cholesterol; TG: triglycerides. The values are presented as the means ± SD. The values in the parenthesis are minimum to maximum.

**Table 2:** Characteristics of the patients with renal biopsy and the control subjects (at baseline).

	*n*	Age (years)	sCr (mg/dL)	eGFR (mL/min/1.73 m^2^)	Proteinuria (g/day)	SBP (mmHg)	DBP (mmHg)
Control	10	51±5	-	-	-	-	-
MN	3	66±11	1.2±0.4	57.1±22.2	4.9±3.4	145±10	92±2
MC	3	39±18	0.7±0.2	86.1±34.2	6.0±3.2	111±10	72±2
Crescentic GN	5	64±10	1.2±0.4	53.4±22.4	0.5±0.5	126±11	75±11
IgA N	15	40±16	0.8±0.4	79.0±24.1	0.6±0.6	127±19	81±12
Lupus N	3	43±10	1.0±0.4	55.9±21.1	1.1±0.3	140±20	82±13
Diabetic N	2	65±1	1.0±0.2	59.8±14.3	3.1±1.0	147±9	68±10
Nephrosclerosis	3	65±2	0.6±0.1	88.2±25.8	0.1±0.0	144±5	76±20
Others	11	34±14	0.8±0.3	87.4±29.0	2.3±3.8	113±22	71±16

Abbreviations: DBP, diastolic blood pressure; eGFR, estimated glomerular filtration rate; GN, glomerulonephritis; MC, minimal change; MN, membranous nephropathy; N, nephropathy; SBP, systolic blood pressure; sCr, serum creatinine. The values are expressed as the means ± SD.

### Correlations between the plasma and urinary levels of VASH-1 and various clinical/histological parameters

**Figure 1:**
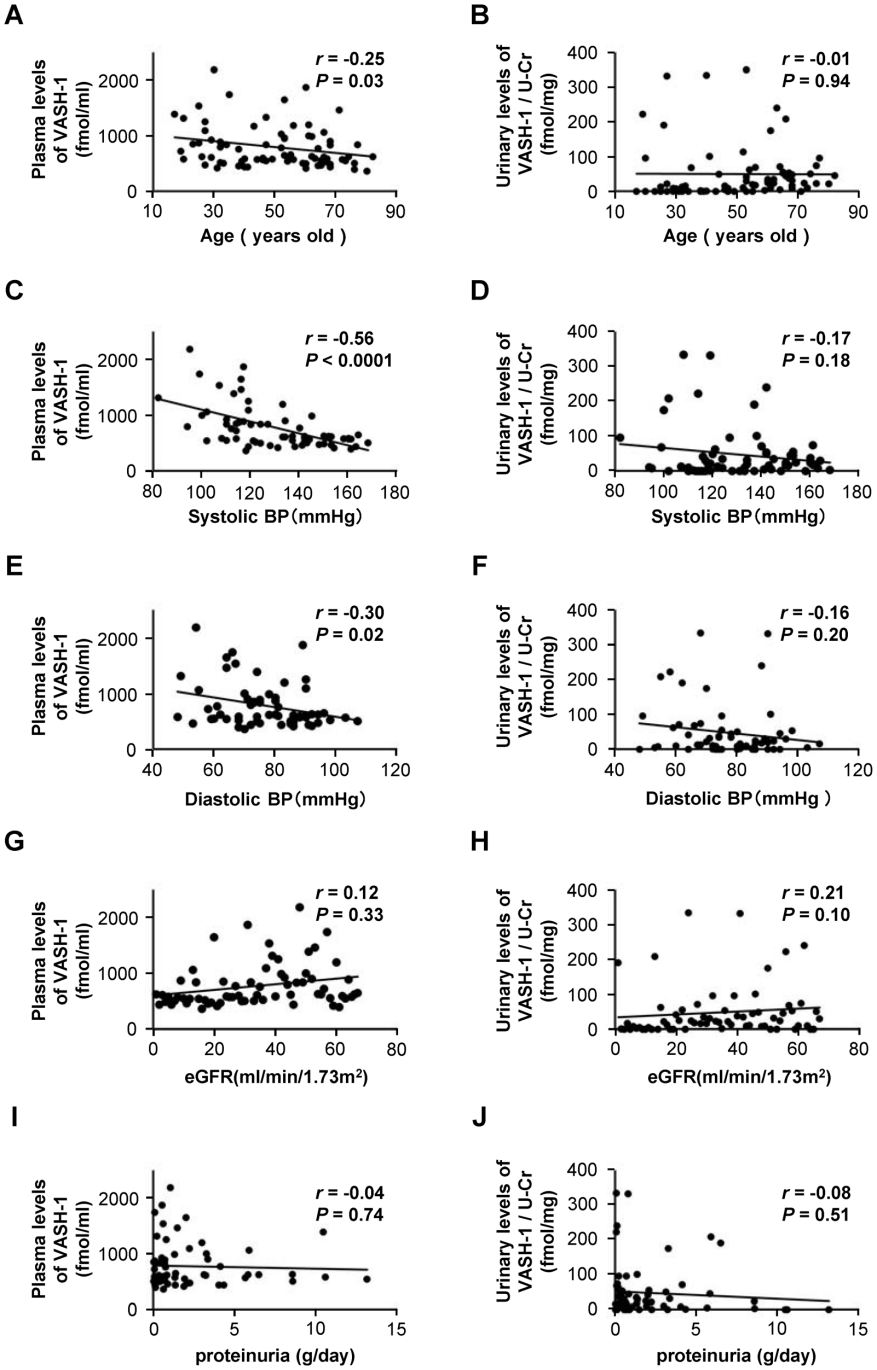
Correlations between the plasma/urinary levels of vasohibin-1 and the clinical parameters. (A) Correlations between the plasma/urinary levels of VASH-1 and age. (B) Correlation between the urinary VASH-1/Cr ratio and age. (C) Correlation between the plasma level of VASH-1 and systolic blood pressure. (D) Correlation between the urinary VASH-1/Cr ratio and systolic blood pressure. (E) Correlation between the plasma level of VASH-1 and diastolic blood pressure. (F) Correlation between the urinary VASH-1/Cr ratio and diastolic blood pressure. (G) Correlation between the plasma level of VASH-1 and eGFR. (H) Correlation between the urinary VASH-1/Cr ratio and eGFR. (I) Correlation between the plasma level of VASH-1 and proteinuria. (J) Correlation between the urinary VASH-1/Cr ratio and proteinuria. Abbreviations: BP, blood pressure; eGFR, estimated glomerular filtration rate; U-Cr, urinary level of creatinine; VASH-1, vasohibin-1.

The plasma levels of VASH-1 detected using enzyme-linked immunosorbent assay (ELISA) were found to be inversely correlated with age and systolic/diastolic blood pressure (SBP/DBP) (Figure 1, panels A, C and E) but not with eGFR or daily proteinuria (Figure 1, panels G and I). The ratio of the urinary level of VASH-1 to creatinine (urinary levels of VASH-1) were not significantly correlated with age, SBP, DBP, eGFR or daily proteinuria (Figure 1, panels B, D, F, H and J). There were no significant correlations between the plasma and urinary levels of VASH-1 (data not shown). There were also no significant correlations between the plasma/urinary levels of VASH-1 and other clinical parameters (gender, body mass index, hemoglobin, HbA1c, fasting plasma glucose, total cholesterol, low-density lipoprotein cholesterol, high-density lipoprotein cholesterol and triglycerides) (data not shown).

**Figure 2:**
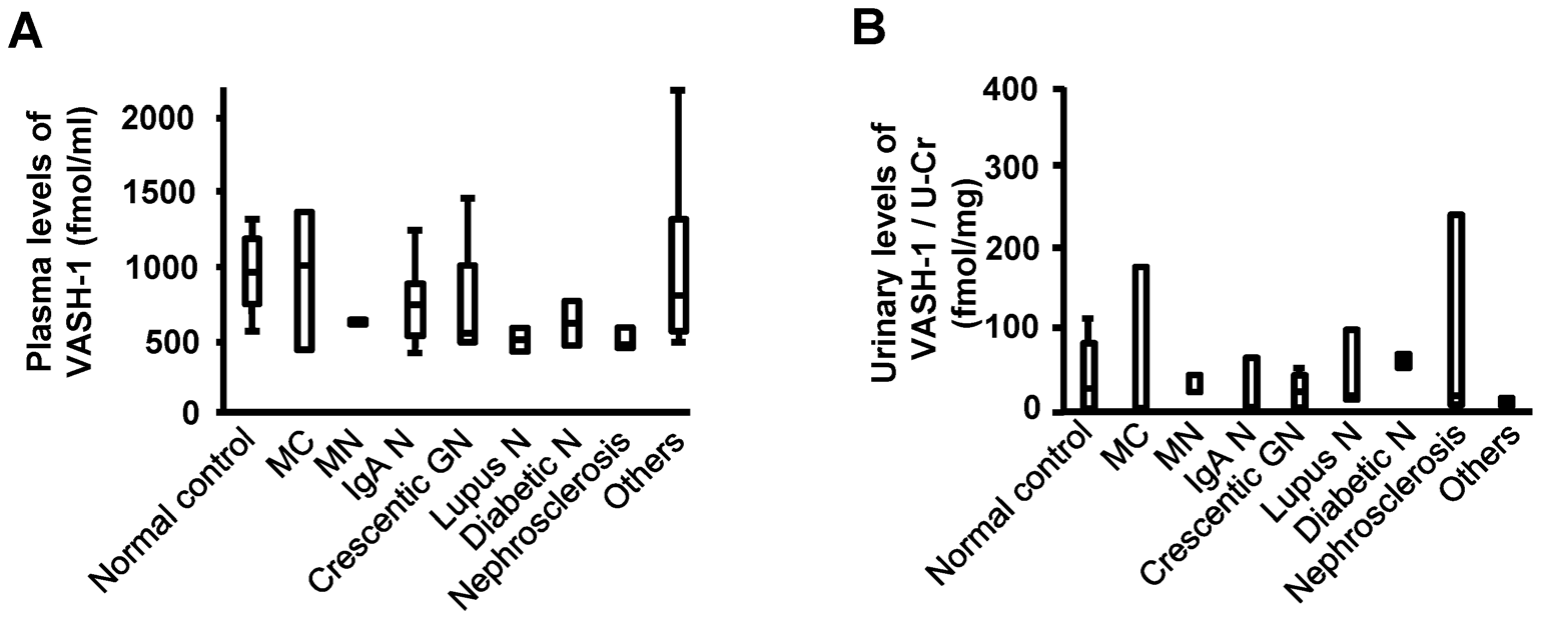
Plasma/urinary levels of vasohibin-1 in patients with various renal disorders. (A) The plasma levels of VASH-1 in patients with various renal disorders. (B) The urinary VASH-1/Cr ratios in patients with various renal disorders. Abbreviations: GN, glomerulonephritis; MC, minimal change; MN, membranous nephropathy; N, nephropathy; U-Cr, urinary level of creatinine; VASH-1, vasohibin-1.

The plasma levels of VASH-1 were positively correlated with the presence of crescent formation (Table 3). There were no significant differences in the plasma/urinary levels of VASH-1 among the patients with various renal disorders (Figure 2).

**Table 3:** Correlations between the plasma and urinary levels of vasohibin-1, and the histological parameters.

	Plasma levels of VASH-1 (fmol/mL)		Urinary levels of VASH-1/U-Cr (fmol/mg)	
	*r*	*P*	*r*	*P*
Mesangial hypercellularity	−0.01	0.92	0.02	0.93
Mesangial sclerosis	−0.08	0.60	0.03	0.87
Crescent formation	0.38	0.01^a^	−0.01	0.96
Global sclerosis	−0.15	0.31	−0.08	0.66
Interstitial cell infiltration	−0.02	0.92	0.04	0.81
Interstitial fibrosis	−0.17	0.28	0.00	0.98
Tubular atrophy	−0.23	0.13	0.02	0.90
Arteriosclerosis	0.15	0.33	0.10	0.58

Abbreviations: U-Cr, urinary level of creatinine; VASH-1, Vasohibin-1.

a *P*<0.05.

### Correlations between the plasma/urinary levels of the VASH-1-SVBP complex and SVBP and various clinical/histological parameters

The plasma levels of the VASH-1-SVBP complex were positively correlated with the plasma levels of VASH-1. The urinary levels of the VASH-1-SVBP complex were positively correlated with the presence of mesangial hypercellularity and crescent formation and the urinary levels of VASH-1. The plasma and urinary levels of SVBP were inversely correlated with age (Table 4).

**Table 4:** Correlations between the plasma and urinary levels of vasohibin-1-small vasohibin-binding protein complex and small vasohibin-binding protein, and the clinical/histological parameters and the plasma/urinary levels of vasohibin-1.

	Plasma levels of VASH-1-SVBP complex (fmol/mL)		Urinary levels of VASH-1-SVBP complex (fmol/mg)		Plasma levels of SVBP (fmol/mL)		Urinary levels of SVBP (fmol/mg)	
	*r*	*P*	*r*	*P*	*r*	*P*	*r*	*P*
Age	−0.11	0.34	−0.12	0.31	−0.25	0.03^a^	−0.32	0.004^b^
Hemoglobin	−0.02	0.87	0.09	0.46	0.13	0.28	0.08	0.54
eGFR	0.01	0.93	0.16	0.20	0.06	0.62	0.14	0.26
Mesangial hypercellularity	−0.15	0.32	0.60	<0.0001^b^	−0.05	0.74	0.17	0.28
Mesangial sclerosis	−0.15	0.33	0.10	0.53	−0.08	0.62	−0.03	0.83
Crescent formation	0.17	0.25	0.32	0.03^a^	−0.04	0.81	0.14	0.35
Global sclerosis	−0.02	0.88	−0.16	0.30	−0.02	0.90	−0.16	0.30
Interstitial cell infiltration	0.05	0.74	0.14	0.37	0.14	0.35	0.21	0.17
Interstitial Fibrosis	−0.09	0.54	0.07	0.65	−0.02	0.90	−0.01	0.94
Tubular atrophy	−0.16	0.30	0.11	0.49	0.03	0.84	−0.03	0.82
Arteriosclerosis	0.19	0.22	−0.16	0.30	0.11	0.46	−0.06	0.70
Plasma levels of VASH-1 (fmol/mL)	0.54	<0.0001^b^	−0.02	0.88	0.16	0.16	−0.10	0.37
Urinary levels of VASH-1/U-Cr (fmol/mg)	−0.004	0.97	0.33	0.003^b^	0.16	0.16	0.11	0.34

Abbreviations: eGFR, estimated glomerular filtration rate; SVBP, small vasohibin-binding protein; U-Cr, urinary level of creatinine; VASH-1, vasohibin-1.

a *P*<0.05.

b *P*<0.01.

### Correlation between VASH-1 and renal outcomes

**Figure 3:**
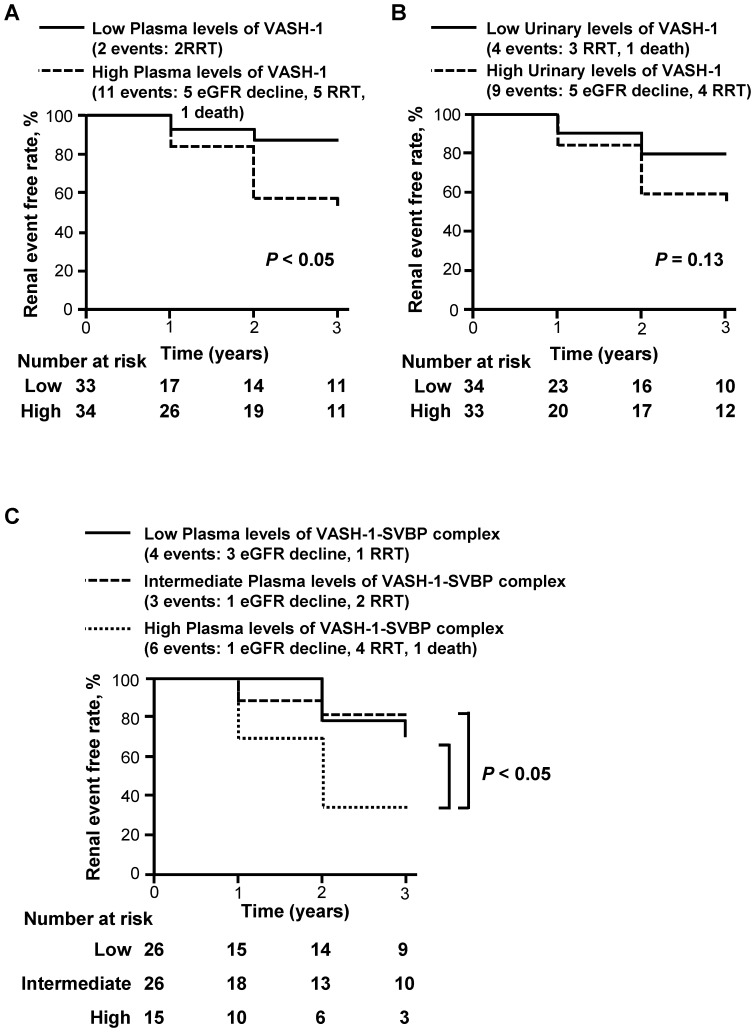
The results of the Kaplan-Meier analysis of the composite renal endpoint. A composite renal event was defined as a decline in the eGFR of more than 30% of the baseline value, initiation of renal replacement therapy or death associated with a renal disorder. In order to perform a Kaplan-Meier analysis of the plasma and urinary levels of VASH-1, the patients were stratified into two groups using the median (609 fmol/mL for the plasma level and 21 fmol/mg for the urinary level of VASH-1) as the cutoff point. The log-rank test was used to compare differences between the two groups. (A and B) Increased plasma levels of VASH-1 were significantly correlated and the urinary levels of VASH-1 tended to be correlated with worse renal outcomes. (C) In order to perform a Kaplan-Meier analysis of the plasma levels of the VASH-1-SVBP complex, the patients were stratified into three groups (<316, 316 to 408 and >408 fmol/mL). The group with the highest plasma levels of the VASH-1-SVBP complex exhibited significantly worse renal outcomes than the groups with moderate or low plasma levels. Abbreviations: eGFR, estimated glomerular filtration rate; RRT, renal replacement therapy, SVBP, small vasohibin-binding protein; VASH-1, vasohibin-1.

The plasma and urinary levels of VASH-1 and the plasma levels of the VASH-1-SVBP complex were inversely correlated with the annual change rates in eGFR, with statistical significance (Table 5). The annual change rates were evaluated as follows: the annual change in eGFR was divided by the eGFR at baseline. We next performed a Kaplan-Meier analysis to evaluate the potential clinical usefulness of the plasma and urinary levels of VASH-1 and the plasma levels of the VASH-1-SVBP complex in predicting poor renal outcomes. The patients were stratified into two or three groups for the Kaplan-Meier analysis. The cutoff values (median) for the plasma and urinary levels of VASH-1 were set at 609 fmol/mL and 21 fmol/mg, respectively, and three groups (<316, 316 to 408 and >408 fmol/mL) were used for the Kaplan-Meier analysis of the plasma levels of the VASH-1-SVBP complex, as shown in Figure 3. We defined a composite renal event as an eGFR decline of greater than 30% from baseline, the initiation of renal replacement therapy or kidney disease-related death. The patients with elevated plasma levels of VASH-1 exhibited a significantly higher incidence of composite renal events and odds ratio than the patients with lower plasma levels of VASH-1 (Figure 3, panel A; Table 6). The patients with elevated urinary levels of VASH-1 also tended to experience a higher incidence of renal events and tended to show a lower odds ratio than the patients with lower urinary levels of VASH-1 (Figure 3, panel B; Table 6). The patients with the highest plasma levels of the VASH-1-SVBP complex exhibited a significantly higher incidence of composite renal events and odds ratio than the patients with the intermediate or the lowest plasma levels of the VASH-1-SVBP complex (Figure 3, panel C; Table 6). The changes in the eGFR and the number of the events in the overall patients and patients with classified renal disorders (diagnosed by renal biopsy) during the three-year follow-up are shown in Table 7. The clinical data for the overall patients and patients with classified renal disorders (diagnosed by renal biopsy) at the final follow-up (after three years) are shown in Tables S1 and S2 in File S1.

**Table 5:** Correlations between the plasma and urinary levels of vasohibin-1, the vasohibin-1-small vasohibin-binding protein complex and small vasohibin-binding protein, and the annual change rates in the estimated glomerular filtration rate.

	Plasma levels of VASH-1 (fmol/mL)		Urinary levels of VASH-1/U-Cr (fmol/mg)		Plasma levels of VASH-1-SVBP complex (fmol/mL)		Urinary levels of VASH-1-SVBP complex (fmol/mg)		Plasma levels of SVBP (fmol/mL)		Urinary levels of SVBP (fmol/mg)	
	*r*	*P*	*r*	*P*	*r*	*P*	*r*	*P*	*r*	*P*	*r*	*P*
Annual change rates in eGFR	−0.33	0.04^a^	−0.46	0.02^a^	−0.36	0.03^a^	0.05	0.77	−0.04	0.80	0.19	0.27

Abbreviations: eGFR, estimated glomerular filtration rate; SVBP, small vasohibin-binding protein; U-Cr, urinary level of creatinine; VASH-1, vasohibin-1. The annual change rates in eGFR were evaluated as follows: the annual change in eGFR was divided by the eGFR at baseline.

a *P*<0.05.

**Table 6:** Results of the multivariate logistic analysis of the risk of composite renal events.

Plasma levels of VASH-1 (fmol/mL)	OR (95% Cl)	*P*-value
Model 1		
<609	1 (referent)	
≥609	9.6 (1.5, 104)	0.02
Model 2		
<609	1 (referent)	
≥609	7.9 (1.1, 95)	0.04
Urinary levels of VASH-1/U-Cr (fmol/mg)		
Model 1		
<21	1 (referent)	
≥21	0.39 (0.03, 3.4)	0.41
Model 2		
<21	1 (referent)	
≥21	0.23 (0.01, 2.7)	0.26
Plasma levels of VASH-1-SVBP complex (fmol/mL)	OR (95% Cl)	*P*-value
Model 1		
<316	0.02 (0.0, 0.3)	0.003
316 to 408	0.01 (0.0, 0.2)	0.001
>408	1 (referent)	
Model 2		
<316	0.02 (0.0, 0.4)	0.01
316 to 408	0.01 (0.0, 0.2)	0.002
>408	1 (referent)	

Abbreviations: CI, confidence interval; eGFR, estimated glomerular filtration rate; OR, odds ratio; SVBP, small vasohibin-binding protein; U-Cr, urinary level of creatinine; VASH-1, vasohibin-1. A composite renal event was defined as a decline in the eGFR of more than 30% of the baseline value, initiation of renal replacement therapy or death associated with a renal disorder. In order to perform a multivariate logistic analysis of the plasma and urinary levels of VASH-1, the patients were stratified into two groups using the median value as the cutoff point, and the plasma levels of the VASH-1-SVBP complex, the patients were stratified into three groups.

Model 1: adjusted for age and gender.

Model 2: adjusted for age, gender and systolic blood pressure.

**Table 7:** The changes in the estimated glomerular filtration rate and the number of events in the overall patients and patients with classified renal disorders.

	Baseline		One year		Two years		Three years		Number of events	
	*n*	eGFR	*n*	eGFR	*n*	eGFR	*n*	eGFR	Total	RRT or Death
All patients	67	60±35	37	58±31	30	56±30	25	57±30	13	8
Patients without RB	22	28±23^a^	13	31±23^a^	11	31±23^a^	10	38±28^a^	10^a^	7^a^
Patients with RB	45	75±29	24	74±24	19	71±21	15	71±21	3	1
MN	3	57±22	2	35±24	1	50	1	44	1	0
MC	3	86±34	3	80±21	2	85±27	1	64	0	0
Crescentic GN	5	53±22	3	62±30	3	51±24	1	65	1	1
IgA N	15	79±24	8	81±16	6	75±6	4	74±9	0	0
Lupus N	3	56±21	2	59±42	2	60±39	2	57±50	0	0
Diabetic N	2	60±14	1	59	1	51	1	43	1	0
Nephrosclerosis	3	88±26	1	94	1	94	1	89	0	0
Others	11	87±29	4	86±18	3	86±18	4	90±18	0	0

Abbreviations: eGFR, estimated glomerular filtration rate (mL/min/1.73 m^2^); GN, glomerulonephritis; MC, minimal change; MN, membranous nephropathy; N, nephropathy; RB, renal biopsy; RRT, renal replacement therapy. A composite renal event was defined as a decline in the eGFR of more than 30% of the baseline value, initiation of RRT or death associated with a renal disorder. The values are expressed as the means ± SD.

a *P*<0.01 versus patients with RB.

The characteristics of the patients classified by the plasma and urinary levels of VASH-1 and the plasma levels of the VASH-1-SVBP complex at baseline and at the final follow-up (after three years) are shown in Tables S3 and S4 in File S1. The follow-up data regarding the changes in the eGFR and the number of the events during the three years of follow-up in the patients classified by the plasma and urinary levels of VASH-1 and the plasma levels of the VASH-1-SVBP complex are shown in Table S5 in File S1. At baseline, the SBP was significantly lower in the group with elevated plasma levels of VASH-1 compared with the group with lower plasma levels of VASH-1 (Table S3 in File S1). Similarly, the SBP was significantly lower in the group with elevated plasma levels of VASH-1-SVBP complex compared with lower plasma levels (Table S3 in File S1). The group with elevated urinary levels of VASH-1 was older and exhibited lower renal function compared with the group with lower levels at both baseline and at all of the follow-up examinations (Tables S3, S4 and S5 in File S1).

The renal function was worse at every time point examined, and the number of composite renal events was greater in patients without a renal biopsy compared with patients who underwent a renal biopsy (Table 7). Most of the patients without renal biopsies were at CKD stage G3b to G5 at baseline. On the other hand, most of the patients who underwent renal biopsies were at CKD stage G1 to G3a at baseline (Table S6 in File S1). The characteristics of the patients with or without renal biopsies classified by the plasma levels of VASH-1 at baseline and at the final follow-up are shown in Table 8 and Table S7 in File S1, respectively. In the patients who underwent a renal biopsy, the group with elevated plasma levels of VASH-1 exhibited preserved renal function, a younger age and lower SBP at the baseline examination (Table 8) and a lower SBP after three years (Table S7 in File S1) compared with the group with lower levels. In patients who did not undergo a renal biopsy, the group with elevated plasma levels of VASH-1 exhibited lower renal function and an older age compared with the group with lower levels after three years (Table S7 in File S1). In the patients without a renal biopsy, the group with elevated plasma levels of VASH-1 exhibited a significantly higher incidence of composite renal events during three years compared with the group with lower levels (Table 9).

**Table 8:** The baseline characteristics of the patients with or without renal biopsy, classified by the plasma levels of vasohibin-1.

	*n*	VASH-1 (fmol/mL)	Age (years)	sCr (mg/dL)	eGFR (mL/min/1.73 m^2^)	Proteinuria (g/day)	SBP (mmHg)
The plasma levels of VASH-1 in patients with RB							
Low (<609 fmol/mL)	23	516±55	52±15	1.0±0.4	67±28	1.4±2.8	137±17
High (≥609 fmol/mL)	22	1036±417^b^	40±19^b^	0.8±0.3	84±27^a^	2.2±3.0	117±19^b^
The plasma levels of VASH-1 in patients without RB							
Low (<609 fmol/mL)	10	500±89	56±19	2.1±1.3	36±26	3.5±3.7	144±21
High (≥609 fmol/mL)	12	1014±396^b^	60±14	3.6±2.3	20±18	2.0±1.7	129±17

Abbreviations: eGFR, estimated glomerular filtration rate; RB, renal biopsy; SBP, systolic blood pressure; sCr, serum creatinine; VASH-1, vasohibin-1. The values are expressed as the means ± SD.

a *P*<0.05 versus the Low group.

b *P*<0.01 versus the Low group.

**Table 9:** The changes in the estimated glomerular filtration rate and the number of events in the patients with or without renal biopsy, classified by the plasma levels of vasohibin-1.

	Baseline		One year		Two years		Three years		Number of events	
	*n*	eGFR	*n*	eGFR	*n*	eGFR	*n*	eGFR	Total	RRT or Death
The plasma levels of VASH-1 in patients with RB										
Low (<609 fmol/mL)	23	67±28	10	80±22	8	73±19	6	70±26	0	0
High (≥609 fmol/mL)	22	84±27^a^	14	69±25	11	70±24	9	73±22	3	1
The plasma levels of VASH-1 in patients without RB										
Low (<609 fmol/mL)	10	36±26	6	43±28	4	51±26	5	51±32	2	2
High (≥609 fmol/mL)	12	20±18	7	22±9	7	19±13	5	20±13	8^a^	5

Abbreviations: eGFR, estimated glomerular filtration rate (mL/min/1.73 m^2^); RB, renal biopsy; RRT, renal replacement therapy; VASH-1, vasohibin-1. A composite renal event was defined as a decline in the eGFR of more than 30% of the baseline value, initiation of RRT or death associated with a renal disorder. The values are expressed as the means ± SD. The average eGFR did not include the values from the patients who received RRT or died.

a *P*<0.05 versus the Low group.

In patients with or without renal biopsies, the groups with elevated urinary levels of VASH-1 were significantly older compared with the groups with lower VASH-1 levels at baseline (Table S8 in File S1). In patients without renal biopsies, the group with elevated urinary levels of VASH-1 exhibited significantly lower levels of proteinuria compared with the group with lower VASH-1 levels at baseline (Table S8 in File S1). The changes in the eGFR and the number of events in the patients with or without renal biopsies classified by the urinary levels of VASH-1 are shown in Table S9 in File S1, but no statistically significant differences were observed among the groups.

## Discussion

Angiogenesis is involved in physiological processes as well as pathological disorders [15]. Angiogenesis-associated factors are involved in the development of the kidneys [16], [17], [18]. Experimental studies have demonstrated the involvement of an imbalance in angiogenesis-related factors in the progression of CKD [19], [20], [21], [22], [23], [24], [25] and the potential therapeutic effects on CKD achieved by modulating these factors [6], [26], [27], [28], [29], [30], [31], [32], [33].

The clinical usefulness of angiogenesis-associated factors, such as VEGF-A, sVEGFR-1/2 and Angiopoietin-1/2, in CKD patients has recently been demonstrated [34], [35], [36], [37], [38], [39]. There are as yet no reports investigating the clinical role of VASH-1 in CKD patients.

In general, VASH-1 is primarily synthesized and secreted by vascular endothelial cells. An inverse correlation between the plasma levels of VASH-1 and age/blood pressure has been observed in CKD patients. At baseline, the SBP was significantly lower in the groups with elevated plasma levels of VASH-1 or VASH-1-SVBP complex compared with the groups with lower levels. However, the differences in the SBP at baseline were not observed when patients were categorized based on the urinary levels of VASH-1. These results suggest that the production of VASH-1 by vascular endothelial cells might be reduced in elderly and hypertensive patients due to systemic arteriosclerotic alterations accompanied by endothelial cell dysfunction, consistent with recent findings suggesting VASH-1 to be a critical factor for the maintenance of endothelial cells [9].

On the other hand, the plasma and urinary levels of VASH-1 and the plasma levels of the VASH-1-SVBP complex at baseline were inversely correlated with the annual rate of changes in the eGFR. The Kaplan-Meier analysis further indicated that elevated plasma levels of VASH-1 and the VASH-1-SVBP complex at baseline successfully predicted a poor renal outcome. In addition, elevated plasma levels of VASH-1 and the VASH-1-SVBP complex at baseline were the significant determinants of a poor renal outcome in the multivariate analysis.

We next classified the patients into groups based on whether they had undergone a renal biopsy. Most of the patients who had not undergone renal biopsies were at CKD stage G3b-G5 (eGFR<45 mL/min/1.73 m^2^). Among them, the eGFR of the patients with elevated plasma levels of VASH-1 consistently tended to be lower throughout the observational period compared with the group with lower levels of VASH-1. With regard to the composite renal endpoint, a significantly higher incidence was noted in the patients with elevated plasma levels of VASH-1 compared with the group with lower levels of VASH-1.

In the patients who underwent renal biopsies, most of the patients were at CKD stage G1-G3a (eGFR 45 mL/min/1.73 m^2^ or higher). Among them, the group with elevated plasma levels of VASH-1 at baseline was younger and exhibited significantly a higher eGFR compared with the group with lower plasma levels of VASH-1. However, the group with elevated plasma levels of VASH-1 tended to experience an increased number of occurrences of the renal endpoint during the three-year observational period. To further investigate these findings, we classified the patients into groups based on whether they had renal disorders diagnosed by renal biopsy. Since the sample size was not large enough to make comparisons among the various renal disorders, the significance of VASH-1 for each particular renal disorder requires further study.

Similar to the present findings, previous reports had demonstrated that an elevated expression of VASH-1 predicted a worse clinical outcome in patients with cancer [40], [41], [42], [43], [44], [45]. In various experimental disease models, including those of cancer and diabetic nephropathy, the administration of adenoviral vectors encoding VASH-1 results in therapeutic effects [8], [12], [13], [14], [46], [47], [48], [49]. More recent findings have demonstrated the role of VASH-1 in enhancing the stress resistance of endothelial cells [9]. Therefore, we suppose that endogenous VASH-1 is upregulated in a compensatory manner in response to increased disease activities and endothelial cell stress in patients with CKD.

Previous reports have demonstrated the expression of VASH-1 in various organs, with a predominant expression observed in the brain and placenta and, to a lesser extent, in the heart and kidneys [8]. In general, the urinary, rather than the plasma, levels of certain factors are supposed to reflect the renal expression. Determining the origin of VASH-1 in the plasma of CKD patients, i.e., the kidney or other distant organs, requires further investigation.

In this study, the plasma and urinary levels of VASH-1 did not exhibit any statistically significant correlations with the renal function or proteinuria. Considering the relatively large molecular size of VASH-1 (42 kDa), it does not easily pass through the glomerular filtration barrier; thus, the urinary levels of VASH-1 are not directly influenced by the amount of VASH-1 in the plasma. Although podocytes may synthesize VASH-1, the lack of a correlation between proteinuria and the urinary VASH-1 level does not support podocytes as the primary source of the urinary VASH-1. On the other hand, VASH-1 may originate from the endothelial cells of the peritubular capillaries in the interstitium, and diffuse into the tubular lumens, thus influencing the urinary levels of VASH-1. Altered tubular reabsorption of VASH-1 due to tubulointerstitial injury may be the alternative factor influencing the urinary levels of VASH-1. However, these speculations require confirmation in further investigations.

In this study, the plasma and urinary levels of SVBP, a secretory chaperone for VASH-1 [10], were inversely correlated with age but not SBP or DBP (data not shown). To date, there is no information available regarding the link between SVBP and clinical parameters, and the causal relationship between SVBP and age may require further investigation. In addition, the urinary levels of the VASH-1-SVBP complex, but not VASH-1, were significantly correlated with the presence of mesangial hypercellularity and crescent formation. In association with these findings, previous reports demonstrated the anti-inflammatory action of VASH-1 and suppressive effects of VASH-1 on mesangial matrix expansion in experimental diabetic nephropathy models [13], [14]. The present results suggest that the urinary levels of the VASH-1-SVBP complex reflect the disease activity in the relatively acute phase of renal disorders.

There are several limitations in the present study. The sample size was not adequate for making comparisons between the patients with various renal disorders. With regard to the healthy control subjects, information other than the age, gender and VASH-1-related measurements were not available. In addition, the precise mechanisms underlying the relationships between the plasma/urinary levels of VASH-1 and the renal outcomes were not fully clarified. To further elucidate these precise mechanisms, basic research on VASH-1 *in vitro* and *in vivo*, including animal models of renal disorders, is required.

In conclusion, our present results demonstrated for the first time the potential role of the plasma/urinary level of VASH-1 and the plasma level of the VASH-1-SVBP complex in predicting future renal functional deterioration in patients with renal disorders.

## Materials and Methods

### Study population

We studied 67 Japanese hospitalized CKD patients with (n = 45) or without (n = 22) renal biopsy samples and 10 healthy control subjects only assented to the disclosure of their age and gender.

All patients attended the Department of Nephrology at Okayama University Hospital (Okayama, Japan) between the years 2007 and 2009. The histological evaluations of the biopsies were performed by two renal pathologists. To measure the eGFR, the modified MDRD equation for Japanese individuals [50] was used. The medical records from the day of the renal biopsy were evaluated. The protocol of the current study was approved by the Institutional Ethical Review Board of Okayama University Hospital (No. 471, Oct. 23, 2007), and written informed consent was obtained from all subjects.

The patients were followed up annually for three years. We defined a composite renal event as an eGFR decline of greater than 30% from baseline, initiation of renal replacement therapy or kidney disease-related death. GFR categories in CKD were defined by KDIGO 2012 Clinical Practice Guideline for the Evaluation and Management of Chronic Kidney Disease.

### ELISA for VASH-1, SVBP and the VASH-1-SVBP complex

The plasma and urinary concentrations of VASH-1, SVBP and the VASH-1-SVBP complex were determined using sandwich ELISA, as previously described [10], utilizing monoclonal antibodies raised against human VASH-1 (VR1 12E7) for capture and horseradish peroxidase-conjugated anti-human VASH-1 antibodies (VC 12F6) for detection. ELISA of the VASH-1-SVBP complex was performed as previously described [10]. All samples were examined in duplicate, and the mean values of the individual samples were utilized for the statistical analysis.

### Histological analysis

Formalin-fixed, paraffin-embedded sections were stained with periodic acid-Schiff (PAS), periodic acid-methenamine-silver (PAM) or Masson's trichrome for light microscopic observation. Renal histological alterations were semiquantitatively evaluated by scoring. The mean percentage of glomeruli with crescent formation and global sclerosis was also determined. The histological evaluation was performed in a blinded fashion by two investigators, and the results were averaged.

### Statistical analysis

All values are expressed as the mean ± standard deviation (SD). Pearson's correlation coefficients were used to evaluate the association between two continuous variables. The Mann-Whitney U-test was used to compare the levels of VASH-1 in the control subjects and patients with renal disorders. Statistical significance was defined as a *P*-value of <0.05. The composite renal event rates were estimated using the Kaplan–Meier method and compared using the log-rank test. A multivariate logistic analysis was performed to examine the correlations between the plasma and urinary levels of VASH-1 and the plasma levels of the VASH-1-SVBP complex according to variables such as age, gender and systolic blood pressure. All statistical tests were performed using the JMP version 9 software package (SAS Institute Inc. Cary, NC, USA).

## Supporting Information

File S1 **Tables S1–S9.** Table S1. The clinical parameters of the study groups after three years (n = 25). Table S2. The final (after three years) characteristics of the patients with renal biopsy (n = 15). Table S3. The baseline characteristics of the patients classified by the plasma and urinary levels of vasohibin-1 and the plasma levels of the vasohibin-1-small vasohibin-binding protein complex. At baseline, the SBP was significantly lower in the group with elevated plasma levels of VASH-1 compared with the group with lower plasma levels of VASH-1. Similarly, the SBP was significantly lower in the group with elevated plasma levels of VASH-1-SVBP complex compared with lower plasma levels. The group with elevated urinary levels of VASH-1 was older and exhibited lower renal function compared with the group with lower levels at baseline. Table S4. The final (after three years) characteristics of the patients classified by the plasma and urinary levels of vasohibin-1 and the plasma levels of the vasohibin-1-small vasohibin-binding protein complex (n = 25). The group with elevated urinary levels of VASH-1 was older and exhibited lower renal function compared with the group with lower levels at the final follow-up (after three years). Table S5. The changes in the estimated glomerular filtration rate and the number of events in the patients classified by the plasma and urinary levels of vasohibin-1 and the plasma levels of the vasohibin-1-small vasohibin-binding protein complex. The group with elevated urinary levels of VASH-1 exhibited lower renal function compared with the group with lower levels at all of the follow-up examinations. Table S6. The CKD stage of the patients with or without renal biopsy. Most of the patients without renal biopsies were at CKD stage G3b to G5 at baseline. On the other hand, most of the patients who underwent renal biopsies were at CKD stage G1 to G3a at baseline. Table S7. The final (after three years) characteristics of the patients with or without renal biopsy, classified by the plasma levels of vasohibin-1 (n = 25). In the patients who underwent a renal biopsy, the group with elevated plasma levels of VASH-1 exhibited a lower SBP compared with the group with lower levels after three years. In patients who did not undergo a renal biopsy, the group with elevated plasma levels of VASH-1 exhibited lower renal function and an older age compared with the group with lower levels after three years. Table S8. The baseline characteristics of the patients with or without renal biopsy, classified by the urinary levels of vasohibin-1. In patients with or without renal biopsies, the groups with elevated urinary levels of VASH-1 were significantly older compared with the groups with lower VASH-1 levels at baseline. In patients without renal biopsies, the group with elevated urinary levels of VASH-1 exhibited significantly lower levels of proteinuria compared with the group with lower VASH-1 levels at baseline. Table S9. The changes in the estimated glomerular filtration rate and the number of events in patients with or without renal biopsy, classified by the urinary levels of vasohibin-1. No statistically significant differences were observed among the groups.
(DOCX)
