# Supplementary material for: Urinary and Plasma Levels of Vasohibin-1 Can Predict Renal Functional Deterioration in Patients with Renal Disorders
Source: PLoS One. 2014 Jun 10;9(6):e96932. doi: 10.1371/journal.pone.0096932 (PMC4051610; doi:10.1371/journal.pone.0096932)
Supplement: File S1 — Tables S1–S9. Table S1. The clinical parameters of the study groups after three years (n = 25). Table S2. The final (after three years) characteristics of the patients with renal biopsy (n = 15). Table S3. The baseline characteristics of the patients classified by the plasma and urinary levels of vasohibin-1 and the plasma levels of the vasohibin-1-small vasohibin-binding protein complex. At baseline, the SBP was significantly lower in the group with elevated plasma levels of VASH-1 compared with the group with lower plasma levels of VASH-1. Similarly, the SBP was significantly lower in the group with elevated plasma levels of VASH-1-SVBP complex compared with lower plasma levels. The group with elevated urinary levels of VASH-1 was older and exhibited lower renal function compared with the group with lower levels at baseline. Table S4. The final (after three years) characteristics of the patients classified by the plasma and urinary levels of vasohibin-1 and the plasma levels of the vasohibin-1-small vasohibin-binding protein complex (n = 25). The group with elevated urinary levels of VASH-1 was older and exhibited lower renal function compared with the group with lower levels at the final follow-up (after three years). Table S5. The changes in the estimated glomerular filtration rate and the number of events in the patients classified by the plasma and urinary levels of vasohibin-1 and the plasma levels of the vasohibin-1-small vasohibin-binding protein complex. The group with elevated urinary levels of VASH-1 exhibited lower renal function compared with the group with lower levels at all of the follow-up examinations. Table S6. The CKD stage of the patients with or without renal biopsy. Most of the patients without renal biopsies were at CKD stage G3b to G5 at baseline. On the other hand, most of the patients who underwent renal biopsies were at CKD stage G1 to G3a at baseline. Table S7. The final (after three years) characteristics of the patients with or withou [file pone.0096932.s001.docx]

**Table S1.** The clinical parameters of the study groups

after three years (n = 25)

| Age | 52 ± 18 | (23-79) |
| --- | --- | --- |
| Gender (male/female) | 12 / 13 |  |
| BMI (kg/m^2^) | 24 ± 4 | (18-31) |
| SBP (mmHg) | 127 ± 16 | (104-167) |
| DBP (mmHg) | 73 ± 10 | (54-93) |
| Hemoglobin (g/dL) | 13 ± 2 | (10-17) |
| HbA1c (NGSP) (%) | 6.3 ± 0.7 | (5.4-7.6) |
| PPG (mg/dL) | 107 ± 22 | (75-163) |
| T-cho (mg/dL) | 181 ± 40 | (101-285) |
| LDL-C (mg/dL) | 104 ± 40 | (45-195) |
| HDL-C (mg/dL) | 57 ± 19 | (31-93) |
| TG (mg/dL) | 164 ± 99 | (45-427) |
| eGFR (mL/min/1.73m^2^) | 60.3 ± 34.7 | (6.1-132.4) |
| Proteinuria (g/gCr) | 0.7 ± 1.2 | (0.0-4.8) |
| Hypertension (%) | 20 |  |
| Diabetes mellitus (%) | 20 |  |
| Dyslipidemia (%) | 48 |  |

Abbreviations: BMI, body mass index; DBP, diastolic

blood pressure; eGFR, estimated glomerular filtration

rate; HDL-C, high-density lipoprotein cholesterol;

LDL-C, low-density lipoprotein cholesterol; NGSP,

national glycohemoglobin standardization program; PPG,

postprandial plasma glucose; SBP, systolic blood

pressure; T-cho, total cholesterol; TG: triglycerides. The

values are presented as the means ± SD. The values in the

parenthesis are minimum to maximum. The proteinuria

was evaluated as follows: the urinary protein was divided

by the urinary creatinine.

**Table S2.** The final (after three years) characteristics of the patients with renal biopsy (*n* = 15)

|  | ***n*** | **Age (years)** | **sCr (mg/dL)** | **eGFR (mL/min/1.73m^2^)** | **Proteinuria (g/gCr)** | **SBP (mmHg)** | **DBP (mmHg)** |
| --- | --- | --- | --- | --- | --- | --- | --- |
| MN | 1 | 65 | 1.3 | 44 | 0.05 | 126 | 65 |
| MC | 1 | 64 | 0.7 | 64 | 0.09 | - | - |
| Crescentic GN | 1 | 63 | 0.9 | 65 | 0.02 | 128 | 72 |
| IgA N | 4 | 37 ± 10 | 0.8 ± 0.2 | 74 ± 9 | 0.17 ± 0.13 | 120 ± 17 | 77 ± 11 |
| Lupus N | 2 | 47 ± 17 | 1.3 ± 1.0 | 57 ± 50 | - | 118 ± 6 | 75 ± 9 |
| Diabetic N | 1 | 67 | 1.3 | 43 | 0.5 | 142 | 54 |
| Nephrosclerosis | 1 | 66 | 0.7 | 89 | 0.13 | 120 | 68 |
| Others | 4 | 31 ± 2 | 0.8 ± 0.2 | 90 ± 18 | 0.14 ± 0.14 | 105 | 69 |

Abbreviations: DBP, diastolic blood pressure; eGFR, estimated glomerular filtration rate; GN, glomerulonephritis; MC, minimal change; MN, membranous nephropathy; N, nephropathy; SBP, systolic blood pressure; sCr, serum creatinine. The values are expressed as the means ± SD. The proteinuria was evaluated as follows: the urinary protein was divided by the urinary creatinine.

**Table S3.** The baseline characteristics of the patients classified by the plasma and urinary levels of vasohibin-1 and the plasma levels of the vasohibin-1-small vasohibin-binding protein complex

|  | ***n*** | **Age (years)** | **sCr (mg/dL)** | **eGFR (mL/min/1.73m^2^)** | **Proteinuria (g/day)** | **SBP (mmHg)** | **DBP (mmHg)** |
| --- | --- | --- | --- | --- | --- | --- | --- |
| The plasma levels of VASH-1 |  |  |  |  |  |  |  |
| Low (< 609 fmol/mL) | 33 | 53 ± 17 | 1.3 ± 0.9 | 58 ± 31 | 2.0 ± 3.2 | 139 ± 18 | 80 ± 14 |
| High (≥ 609 fmol/mL) | 34 | 47 ± 20 | 1.7 ± 1.9 | 63 ± 39 | 2.1 ± 2.6 | 121 ± 19^b^ | 74 ± 12 |
| The urinary levels of VASH-1 |  |  |  |  |  |  |  |
| Low (< 21 fmol/mg) | 34 | 42 ± 16 | 1.2 ± 1.2 | 69 ± 33 | 2.4 ± 3.4 | 128 ± 20 | 78 ± 14 |
| High (≥ 21 fmol/mg) | 33 | 58 ± 17^b^ | 1.8 ± 1.7 | 52 ± 36^a^ | 1.8 ± 2.2 | 132 ± 21 | 76 ± 13 |
| The plasma levels of the VASH-1-SVBP complex |  |  |  |  |  |  |  |
| Low (< 316 fmol/mL) | 26 | 55 ± 18 | 1.3 ± 0.9 | 60 ± 31 | 1.3 ± 1.9 | 138 ± 18 | 81 ± 12 |
| Intermediate (316 to 408 fmol/mL) | 26 | 48 ± 18 | 1.5 ± 1.4 | 63 ± 38 | 2.5 ± 3.4 | 128 ± 20 | 76 ± 14 |
| High (> 408 fmol/mL) | 15 | 44 ± 19 | 2.1 ± 2.2 | 56 ± 39 | 2.6 ± 3.3 | 120 ± 19^a^ | 72 ± 14 |

Abbreviations: DBP, diastolic blood pressure; eGFR, estimated glomerular filtration rate; SBP, systolic blood pressure; sCr, serum creatinine; SVBP, small vasohibin-binding protein; VASH-1, vasohibin-1. The values are expressed as the means ± SD.

^a^*P* < 0.05 versus the Low group. ^b^*P* < 0.01 versus the Low group.

**Table S4.** The final (after three years) characteristics of the patients classified by the plasma and urinary levels of vasohibin-1 and the plasma levels of the vasohibin-1-small vasohibin-binding protein complex (*n* = 25)

|  | ***n*** | **Age (years)** | **sCr (mg/dL)** | **eGFR (mL/min/1.73m^2^)** | **Proteinuria (g/gCr)** | **SBP (mmHg)** | **DBP (mmHg)** |
| --- | --- | --- | --- | --- | --- | --- | --- |
| The plasma levels of VASH-1 |  |  |  |  |  |  |  |
| Low (< 609 fmol/mL) | 11 | 54 ± 17 | 1.1 ± 0.6 | 61 ± 29 | 0.2 ± 0.1 | 120 ± 8 | 73 ± 8 |
| High (≥ 609 fmol/mL) | 14 | 50 ± 18 | 1.6 ± 1.3 | 54 ± 32 | 1.1 ± 1.5 | 136 ± 18^a^ | 74 ± 12 |
| The urinary levels of VASH-1 |  |  |  |  |  |  |  |
| Low (< 21 fmol/mg) | 10 | 38 ± 12 | 0.9 ± 0.4 | 74 ± 27 | 0.2 ± 0.2 | 125 ± 21 | 76 ± 8 |
| High (≥ 21 fmol/mg) | 15 | 61 ± 14^b^ | 1.7 ± 1.2 | 46 ± 28^a^ | 1.0 ± 1.5 | 128 ± 13 | 72 ± 11 |
| The plasma levels of the VASH-1-SVBP complex |  |  |  |  |  |  |  |
| Low (< 316 fmol/mL) | 11 | 57 ± 18 | 1.4 ± 1.0 | 55 ± 29 | 0.8 ± 1.5 | 122 ± 12 | 69 ± 8 |
| Intermediate (316 to 408 fmol/mL) | 10 | 52 ± 15 | 1.2 ± 0.6 | 53 ± 26 | 0.6 ± 1.0 | 131 ± 20 | 78 ± 11 |
| High (> 408 fmol/mL) | 4 | 37 ± 18 | 1.8 ± 0.5 | 74 ± 46 | 0.4 ± 0.5 | 132 ± 2 | 74 ± 6 |

Abbreviations: DBP, diastolic blood pressure; eGFR, estimated glomerular filtration rate; SBP, systolic blood pressure; sCr, serum creatinine; SVBP, small vasohibin-binding protein; VASH-1, vasohibin-1. The values are expressed as the means ± SD. The proteinuria was evaluated as follows: the urinary protein was divided by the urinary creatinine.

^a^*P* < 0.05 versus the Low group. ^b^*P* < 0.01 versus the Low group.

**Table S5.** The changes in the estimated glomerular filtration rate and the number of events in the patients classified by the plasma and urinary levels of vasohibin-1 and the plasma levels of the vasohibin-1-small vasohibin-binding protein complex

|  | **Baseline** | | **One year** | | **Two years** | | **Three years** | | **Number of events** | |
| --- | --- | --- | --- | --- | --- | --- | --- | --- | --- | --- |
|  | ***n*** | **eGFR** | ***n*** | **eGFR** | ***n*** | **eGFR** | ***n*** | **eGFR** | **Total** | **RRT or Death** |
| The plasma levels of VASH-1 |  |  |  |  |  |  |  |  |  |  |
| Low (< 609 fmol/mL) | 33 | 58 ± 31 | 16 | 66 ± 30 | 12 | 65 ± 26 | 11 | 61 ± 29 | 2 | 2 |
| High (≥ 609 fmol/mL) | 34 | 63 ± 39 | 21 | 53 ± 31 | 18 | 50 ± 32 | 14 | 54 ± 32 | 11 | 6 |
| The urinary levels of VASH-1 |  |  |  |  |  |  |  |  |  |  |
| Low (< 21 fmol/mg) | 34 | 69 ± 33 | 19 | 71 ± 27 | 14 | 71 ± 24 | 10 | 74 ± 27 | 4 | 4 |
| High (≥ 21 fmol/mg) | 33 | 52 ± 36^a^ | 18 | 45 ± 30^a^ | 16 | 43 ± 30^b^ | 15 | 46 ± 28^a^ | 9 | 4 |
| The plasma levels of the VASH-1-SVBP complex |  |  |  |  |  |  |  |  |  |  |
| Low (< 316 fmol/mL) | 26 | 60 ± 31 | 15 | 62 ± 30 | 14 | 57 ± 28 | 11 | 55 ± 28 | 4 | 1 |
| Intermediate (316 to 408 fmol/mL) | 26 | 63 ± 38 | 16 | 63 ± 30 | 12 | 56 ± 30 | 10 | 53 ± 26 | 3 | 2 |
| High (> 408 fmol/mL) | 15 | 56 ± 39 | 6 | 37 ± 34 | 4 | 55 ± 45 | 4 | 74 ± 46 | 6 | 5 |

Abbreviations: eGFR, estimated glomerular filtration rate (mL/min/1.73m^2^); RRT, renal replacement therapy; SVBP, small vasohibin-binding protein; VASH-1, vasohibin-1. A composite renal event was defined as a decline in the eGFR of more than 30% of the baseline value, initiation of RRT or death associated with a renal disorder. The values are expressed as the means ± SD. ^a^*P* < 0.05 versus the Low group. ^b^*P* < 0.01 versus the Low group.

**Table S6.** The CKD stage of the patients with or without renal biopsy.

|  | G1 | G2 | G3a | G3b | G4 | G5 | Total |
| --- | --- | --- | --- | --- | --- | --- | --- |
| The number of patients with RB | 16 | 17 | 5 | 2 | 5 | 0 | 45 |
| The number of patients without RB | 1 | 2 | 2 | 4 | 6 | 7 | 22 |

Abbreviations: CKD, chronic kidney disease; RB, renal biopsy.

**Table S7.** The final (after three years) characteristics of the patients with or without renal

biopsy, classified by the plasma levels of vasohibin-1 (*n* = 25)

|  | ***n*** | **Age (years)** | **sCr (mg/dL)** | **eGFR (mL/min/1.73m^2^)** | **Proteinuria (g/gCr)** | **SBP (mmHg)** |
| --- | --- | --- | --- | --- | --- | --- |
| The plasma levels of VASH-1 in patients with RB |  |  |  |  |  |  |
| Low (< 609 fmol/mL) | 6 | 51 ± 15 | 1.0 ± 0.4 | 67 ± 28 | 1.4 ± 2.8 | 137 ± 17 |
| High (≥ 609 fmol/mL) | 9 | 43 ± 17 | 0.8 ± 0.3 | 84 ± 27 | 2.2 ± 3.0 | 117 ± 19^b^ |
| The plasma levels of VASH-1 in patients without RB |  |  |  |  |  |  |
| Low (< 609 fmol/mL) | 5 | 58 ± 21 | 1.4 ± 0.7 | 51 ± 32 | 0.2 ± 0.1 | 123 ± 7 |
| High (≥ 609 fmol/mL) | 5 | 64 ± 10^b^ | 2.8 ± 1.5 | 20 ± 13^a^ | 2.7 ± 1.6 | 144 ± 18 |

Abbreviations: eGFR, estimated glomerular filtration rate; RB, renal biopsy; SBP,

systolic blood pressure; sCr, serum creatinine; VASH-1, vasohibin-1. The values are

expressed as the means ± SD. The proteinuria was evaluated as follows: the urinary

protein was divided by the urinary creatinine.

^a^*P* < 0.05 versus the Low group. ^b^*P* < 0.01 versus the Low group.

**Table S8.** The baseline characteristics of the patients with or without renal biopsy, classified by the urinary levels of vasohibin-1

|  | ***n*** | **VASH-1 (fmol/mg)** | **Age (years)** | **sCr (mg/dL)** | **eGFR (mL/min/1.73m^2^)** | **Proteinuria (g/day)** | **SBP (mmHg)** |
| --- | --- | --- | --- | --- | --- | --- | --- |
| The urinary levels of VASH-1 in patients with RB |  |  |  |  |  |  |  |
| Low (< 21 fmol/mg) | 26 | 5.8 ± 6.9 | 41 ± 16 | 0.8 ± 0.3 | 78 ± 27 | 1.7 ± 3.1 | 125 ± 19 |
| High (≥ 21 fmol/mg) | 19 | 114 ± 103^b^ | 53 ± 20^a^ | 0.9 ± 0.4 | 73 ± 31 | 1.9 ± 2.5 | 130 ± 22 |
| The urinary levels of VASH-1 in patients without RB |  |  |  |  |  |  |  |
| Low (< 21 fmol/mg) | 8 | 6.7 ± 6.9 | 45 ± 18 | 2.7 ± 2.0 | 36 ± 32 | 4.7 ± 3.4 | 137 ± 20 |
| High (≥ 21 fmol/mg) | 14 | 57 ± 48^b^ | 66 ± 9^b^ | 3.0 ± 2.0 | 24 ± 17 | 1.5 ± 1.6^a^ | 135 ± 20 |

Abbreviations: eGFR, estimated glomerular filtration rate; RB, renal biopsy; SBP, systolic blood pressure; sCr, serum creatinine; VASH-1, vasohibin-1. The values are expressed as the means ± SD.

^a^*P* < 0.05 versus the Low group. ^b^*P* < 0.01 versus the Low group.

**Table S9.** The changes in the estimated glomerular filtration rate and the number of events in patients with or without renal biopsy, classified by the urinary levels of vasohibin-1

|  | **Baseline** | | **One year** | | **Two years** | | **Three years** | | **Number of events** | |
| --- | --- | --- | --- | --- | --- | --- | --- | --- | --- | --- |
|  | ***n*** | **eGFR** | ***n*** | **eGFR** | ***n*** | **eGFR** | ***n*** | **eGFR** | **Total** | **RRT or Death** |
| The urinary levels of VASH-1 in patients with RB |  |  |  |  |  |  |  |  |  |  |
| Low (< 21 fmol/mg) | 26 | 78 ± 27 | 16 | 76 ± 22 | 8 | 71 ± 23 | 8 | 75 ± 9 | 1 | 1 |
| High (≥ 21 fmol/mg) | 19 | 73 ± 31 | 8 | 69 ± 28 | 11 | 71 ± 20 | 7 | 67 ± 8 | 2 | 0 |
| The urinary levels of VASH-1 in patients without RB |  |  |  |  |  |  |  |  |  |  |
| Low (< 21 fmol/mg) | 7 | 36 ± 32 | 3 | 44 ± 41 | 2 | 70 ± 42 | 2 | 70 ± 42 | 3 | 3 |
| High (≥ 21 fmol/mg) | 14 | 24 ± 17 | 10 | 27 ± 15 | 9 | 22 ± 16 | 8 | 27 ± 18 | 7 | 4 |

Abbreviations: eGFR, estimated glomerular filtration rate (mL/min/1.73m^2^); RB, renal biopsy; RRT, renal replacement therapy; VASH-1, vasohibin-1. A composite renal event was defined as a decline in the eGFR of more than 30% of the baseline value, initiation of RRT or death associated with a renal disorder. The values are expressed as the means ± SD.
